# Pericarditis

**Published:** 1830-10-01

**Authors:** 


### XIII.

I. ON THE RECENT IMPROVEMENTS IN THE ART OF DISTINGUISHING THE VARIOUS DISEASES OF THE HEART, BEING THE LUMLEYAN LECTURES DELIVERED BEFORE THE ROYAL COLLEGE OF PHYSICIANS IN THE YEAR 1829. By *John Elliotson*, M.D. Cantab. F.R.S. &c. &c. Folio, pp. 35, double Columns, with Eight Plates. Sept. 1830.

II. CASES OF PERICARDITIS. By *M. Louis*, Physician to LA PITIÉ.

WE believe there is no hospital physician in this metropolis, who labours more strenuously to convert a public institution into an instrument for increasing knowledge and alleviating human sufferings, than the author of the work under review. Besides his excellent translation of Blumenbach's Physiology, Dr. Elliotson has, at various times, favoured his professional brethren with a work on hydrocyanic acid in affections of the stomach—and many papers in the Medico-chirurgical Transactions, especially on antimonial powder—the use of opium in diabetes—on the medical properties of quinine—on subcarbonate of iron in chorea—on the same in tetanus—on sulphate of copper in chronic diarrhœa—on rupture of the stomach—Fallopian pregnancy—glanders communicated from the horse to the human subject, &c. In all these works and insulated memoirs, our author has shewn himself a close observer and an active practitioner, who rarely or never suffers himself to be seduced from useful facts by the allurements of theory or wild speculation. Hence it is that a great practical interest is kept up in the reader's mind, while perusing Dr. Elliotson's papers, which is not the case with the generality of works coming under our notice. There is another point which is deserving of attention. Dr. Elliotson is very scrupulous in the statements which he makes, and highly candid in the results of all his cases. We have no trifling reason to know that inattention to scrupulous veracity and unequivocal candour has been a great draw-back on the progress of medical science.

In an eloquent introduction to the first lecture, Dr. Elliotson draws the attention of his brethren to the means by which improvements are most likely to be effected in the art of medicine—namely, by increasing our knowledge of the nature of diseases—in diagnosis—and therapeutics. The knowledge of diseased structure cannot be acquired without a knowledge of the healthy—that is, anatomy is necessary to pathology: but anatomy alone will give us little insight into disease.

"An anatomist is not necessarily even a physiologist: and Mery was accustomed to say, 'We anatomists are like the porters of Paris, who are well acquainted with all its streets, as well as all its lanes and alleys, but know nothing of what passes within the houses.'" 1.

The structure of the brain would never have led us to know that it was the organ of thought and feeling, without observation of those functions—in other words, physiology. Before proceeding to the immediate subject of this lecture—Pericarditis, we must indulge in an extract or two respecting the utility of auscultation.

"In improving diagnosis, it is impossible to discover only what is obviously useful. The research must be made generally, and what is at once useful and what is not, must turn up together.

In the diseases of other organs, we always aim at accuracy of diagnosis without any hope of utility. When a solid tumour exists in the abdomen, we endeavour to ascertain whether it is the liver, the spleen, the pylorus, an ovarium, a new formation, or whatever else, although the treatment would probably be the same in all.

But there *is* immediate utility in the discoveries of Avenbrugger and Laennec. No one will pretend that the diagnosis in chronic diseases of the chest is, with perhaps the exception of phthisis, generally satisfactory. Before I adopted auscultation, I know that I frequently discovered disease of the heart after death, where I had not previously suspected it; and frequently found the organ sound when I had supposed it diseased. When I was correct in expecting to see organic affection of the heart, I was often wrong as to the precise nature of the lesion. Too often has auscultation at once revealed disease of the heart to me, when by good practitioners no affection of the heart or even of the chest had been suspected, or the case had been named nervous palpitation or asthma; when the lungs had been regarded as the seat of the malady, or the case been treated with the more violent remedies of hydrothorax. Repeatedly have I seen chronic bronchitis with extreme congestion in the lungs mistaken for hydrothorax, and unavoidably so from the omission of percussion and auscultation, because the symptoms were precisely the same, with the exception of those which percussion and auscultation only could disclose. Inflammation of the substance of the lungs takes place continually during other diseases without being obvious before death to any but the auscultator and percussor. Without the aid of the ear, who can ever distinguish emphysema of the lungs, or in every case pneumatothorax. Both may be readily mistaken for hydrothorax. The symptoms may be pallid face, with purple lips, orthopnœa, sudden starting from sleep to the waking state, a small and intermitting pulse, cold extremities, and swollen feet. The remedies of hydrothorax may appear indicated; but the ear will show the chest to sound hollow, and far too hollow, at its very lowest parts, while there is in the first case little, and in the second no, respiratory murmur at the very place where the hollow sound is heard. Nothing but the ear can show the nature of these cases; nothing but the ear could distinguish them from each other,—without the ear, no case can be known with certainty as hydrothorax, however marked the symptoms." 5.

Dr. E. properly observes that the study of auscultation can never justify the slightest neglect of general symptoms and history of disease. For our own parts, we firmly believe that those who study auscultation, take more pains in observing symptoms than those who go on in the old routine. It is curious that the celebrated Hook, in his "method of improving natural philosophy," has prophesied the stethoscope.

" 'There may be a possibility,' says he, 'of discovering the internal motions and actions of bodies by the sound they make; who knows but that as in a watch we may hear the beating of the balance, and the running of the wheels, and the striking of the hammers, and the grating of the teeth, and multitudes of other noises; who knows, I say, but that it may be possible to discover the motions of the internal parts of bodies, whether animal, vegetable, or mineral, by the sound they make; that one may discover the works performed in the several offices and shops of a man's body, and thereby discover what engine is out of order, what works are going on at several times, and lie still at others, and the like."

And again he says:—" 'but yet again, I have this encouragement, not to think all these things utterly impossible, though never so much derided by the generality of men, and never so seemingly mad, foolish, and fantastic, that as the thinking them impossible cannot much improve my knowledge, so the believing them possible may perhaps be an occasion for taking notice of such things as another would pass by without regard as useless. And somewhat more of encouragement I have also from experience, that I have been able to hear very plainly the beating of a man's heart, and 'tis common to hear the motion of the wind to and fro in the guts and other small vessels; the stopping in the lungs is easily discovered by the wheezing.' " 6.

Passing over many judicious and learned remarks, we come to—

### DISEASES OF THE PERICARDIUM.

PERICARDITIS. This is a disease in which auscultation is less to be depended on than in many others. The anatomical characters differ little from those of other inflammations of serous membranes. The pericardium was never found thickened by Laennec—though Baillie says he has often found it so. Perhaps the layers of coagulable lymph, which are often deposited on the membrane, may have been considered by Baillie as thickening. The serum effused, is far less in proportion to the fibrine than in pleurisy. It seldom amounts to a pint—sometimes none is found. It is occasionally of a whitish lemon-colour—rarely limpid—sometimes very turbid, or absolutely curdled. It has been found bloody, and even sero-purulent. If the patient survive the acute stage, absorption takes place, and the two surfaces of the pericardium become more or less agglutinated by the intervention of the fibrine. The adhesions are sometimes very thick and fibrous—or even cartilaginous.

"I have frequently seen the whole cardiac and parietal pericardium coherent, (and even the proper auricles concreted to the ventricles) so that no pericardial cavity existed, the serum being entirely absorbed, and the fibrine nearly so: and such cases have occasionally been mistaken for instances of the absence of the pericardium.

Both when the pericardium is very coherent, and when it is only thickened, the morbid action may be more intense in particular spots; for we often find knobs of cartilage at different parts, some of which dip deep into the substance of the heart.

Sometimes the fibrine effused becomes cellular, and, contracting no adhesions, lies pale, like lace, upon the surface of the heart; sometimes merely an opaque white patch remains, which can be peeled off; and sometimes, instead of smooth patches, we have opaque white granulations.

If the inflammation has been severe, lymph is often found in more or less quantity on even the external surface of the pericardium, uniting it by bands to the pleura.

The reason is not obvious why the fibrine within the pericardium sometimes adheres, and sometimes contracts no adhesions. The degree of serous effusion and consequent proportionate separation of parts affords no explanation, because, first, we often see one portion of lymph adherent, while another by its side is not; and secondly, there is often a total absence of adhesion without sufficient serous effusion to account for it.

The substance of the heart after pericarditis may be unchanged, or redder or paler than usual, yellowish or brown, hardened or softened. After the chronic disease it has been found hypertrophied. When the organ is softened it usually is that we observe the effused serum to be bloody. In the disease of softening of the heart I have almost always found bloody serum in the pericardium."

The causes of pericarditis are those of other inflammations—the most common being exposure to cold when the body has been heated, more especially if rheumatism be at the same time induced. It will often occur simultaneously with rheumatism, or not till after it has declined or altogether ceased. It is sometimes the result of a direct metastasis of rheumatism from the joints.

"If pericarditis becomes chronic, and it is usually not very violent but really disposed to assume the chronic form, and frequently steals on as a chronic disease from the abuse of fermented liquors, the valves and finally the substance of the heart, as I have mentioned, become diseased. From these circumstances, the connexion between rheumatism and affection of the heart was first noticed in the stage of organic disease, and rheumatism said to produce not inflammation but disease of the heart. Occasionally, the pericardium may not be affected, and occasionally but in a secondary manner: yet of this I am certain, that nearly all the cases of affections of the heart, after rheumatism, are originally pericarditis, and that, when the inner membrane is thus affected from the first, so also is the pericardium. Among the cases of organic disease of the heart connected with rheumatism, published by Dr. Wells nearly twenty years ago, in the Transactions of a Society for the Improvement of Medical and Chirurgical Knowledge, those which proved fatal displayed a complete abolition of the pericardial cavity, or strong or abundant partial adhesions, and those which did not prove fatal were marked by decided symptoms of pericarditis. In nearly all those mentioned by Sir David Dundas, in the first volume of the Medico-Chirurgical Transactions of London, the pericardium was adherent. Every dissection that I have made after death, during the early period of the disease, has proved the case to be violent pericarditis; the history of every chronic case that I have witnessed could be clearly traced back to pericarditis; and every the least affection of the heart that I have seen take place during rheumatism has been marked pericarditis. The pleura, particularly of the left side, is occasionally inflamed at the same time, and the subsequent chronic organic disease of the heart is of every possible variety. Dr. Pitcairn, of St. Bartholomew's Hospital, was the first who noticed, in about 1788, the connexion of rheumatism with disease of the heart; and Dr. Baillie, in 1797, was the first who published on the subject. They considered the disease to be a morbid growth of the heart. Sir David Dundas, who published many years afterwards,—in 1808, upon the subject, without reference to the observations of these physicians, and asserting his belief that no account of the matter was to be found in any medical writer, (as though, remarks Dr. Wells, it was easy to suppose him ignorant of what had been published twelve years before in so popular a work as Dr. Baillie's morbid anatomy,) mentions the disease as dilatation of the heart, and chiefly of the left ventricle, with paleness and softness of its substance, and adherence of the pericardium. But from the imperfection of morbid anatomy in this country twenty years ago, his description is very loose. However, in one of Dr. Wells's cases, which proved fatal early, and was opened by Mr. Brodie, nothing but pericarditis was discoverable; and Dr. Wells, no less distinguished for his sagacity than his independence, evidently regarded the rheumatic affection of the heart as inflammatory, by advising copious bleeding in the outset." 9.

The disease, observes our author, appears sometimes to remain long in the form of mere pericarditis—or at least an inflammatory affection—sometimes continuing for years without signs of organic disease, proving troublesome only when cold is caught, and a fresh attack of articular rheumatism is induced. Most instances of rheumatic pericarditis commence in young persons, from about the age of puberty to near thirty. Occasionally we see it in the younger—rarely for the first time in the older. Our author once saw it in an infant.

The French do not appear to be aware of the connexion of pericarditis with rheumatism, except as an ordinary instance of internal inflammation upon the sudden retrocession of an external disease. The following symptomatology and diagnostic marks must be given in the words of the able author.

" Acute pericarditis is of course attended by more or less pyrexia. There is a pain in the region of the heart, sometimes severe and lancinating: generally darting through to the left scapula, upwards to the left clavicle and shoulder, and down the arm a certain way, and, what is remarkable, rarely extending quite so far as the elbow. I lately had a case in which the pain extended down the fore arm, but it did not quite reach the wrist. The pain is increased by pressing forcibly upon or between the ribs and cartilages over the heart, and by pressing with the points of the fingers upwards against the diaphragm, under the cartilages of the left false ribs, frequently even by pressing the epigastrium and left hypochondrium in the usual manner. The pain is often increased on inspiration, and by lying on the left side. I think patients are usually easiest upon their back. The respiration is rapid, but less so than in affections of the lungs. There is sometimes a cough, which is dry. Nearly always palpitation, frequently violent, at least upon exertion. Sometimes, though more rarely, a disposition to syncope. The pulse varies exceedingly. It is necessarily quick; and often, but not always, small, in proportion to the heart's action; and only sometimes intermittent and irregular; neither is it always hard or even very full. The countenance is described as anxious, and the features contracted: but this I imagine happens only when the pain is acute, and is equally the case in pleuritis.

On examination by the ear, the *whole* heart is found acting more forcibly, and with a clearer sound, than in health. But this is all. Auscultation appears to me, however, of negative use. We do not discover the loud murmur nor the sonorous or sibilous rattle of bronchitis, the crepitous rattle or obscure respiratory murmur of pneumonia, nor the œgophony of pleuritic effusion, unless these diseases are combined with pericarditis. Neither have we the partially excessive or defective impulse or sound, or preternatural sounds, of organic diseases of the heart. In all uncombined cases therefore, light is thrown on the disease. I remember once having found auscultation of great use in the diagnosis of a disease which might have been considered clearly pericarditis. The patient was a poor Irishman, and the Irish are by no means happy in their attempts at lucid history and description of their diseases. He complained of pain in the region of the heart, increased on pressure, palpitation, dyspnœa, and declared he had been ill but a few days. The case appeared pericarditis. The pulse was full, and the constitution good. There appeared every reason to bleed him freely, and put the whole antiphlogistic plan in force. But on listening to the heart's action, the left ventricle gave a violent dead noiseless blow against the chest, and the case was evidently one of hypertrophy of the left ventricle. I insisted to the man that he had long been ill, and it was ascertained from his own mouth, and from his wife, that he had suffered palpitation and dyspnœa for a great length of time, and that the error of his history arose from his having been compelled to leave off work for a few days only before. He died in a fortnight, and great hypertrophy of the left ventricle was discovered.

M. Collier says that the action of the heart is accompanied by a sound resembling that of new leather. Laennec does not mention it, but remarks the occasional occurrence of a sort of click, which some persons mistake for a *bruit de soufflet*.

The diagnosis of pericarditis is thought by many to be extremely difficult. Laennec declares that he has frequently suspected it where it was not found, and found it where he had not suspected it. By close inquiry into the existence of all the marks just mentioned, I confess the diagnosis has never proved difficult to me. I would particularly lay stress upon the extension of the pain from the region of the heart to the scapula, shoulder, and a certain way down the arm—symptoms which patients will not always mention unless questioned respecting them; and its increase on strong pressure upon or between the ribs and cartilages over the heart, and upwards under the cartilages of the left false ribs. These two points I do not remember to have seen mentioned any where, and the others are not dwelt upon in some of the best books. In Andral's *Clinique Medicale*, pain of the epigastrium on pressure is said to have occurred in some cases, but the point is not spoken of as if inquired into; in one case only is the extension of pain to the arm mentioned; and its extension even to the shoulder does not seem to have formed an object of inquiry.

I am certain that, by a scrutinizing examination, the existence of pericarditis will very rarely be mistaken; and from this conviction, and the frequency of its occurrence during acute rheumatism, I make it as invariable a rule to examine the cardiac region by the touch and hearing in every case of acute rheumatism, as the usual seats of hernia are examined by us all in cases of colic and intestinal inflammation. Were this rule universally observed, practitioners would not be occasionally surprised by the death of patients in what had been considered merely acute rheumatism." 10.

We shall here introduce two or three cases of pericarditis recently published by M. Louis as occurring in La Pitié, and in which some phenomena are recorded, and some opinions broached, which are deserving of notice.

Case 1. A young man, 25 years of age, had been ill 15 days before admittance into St. Raphael's ward, La Pitié. The complaint commenced with dyspnœa, accompanied by pain in the left side, immediately after receiving a fall while carrying a burthen, in which fall he struck against the pole of a voiture. The dyspnœa and pain increased—palpitations ensued—the appetite diminished—and the man was obliged to leave off work. Four days after the accident, his sleep became disturbed with frightful dreams and startings up in bed—diarrhœa came on, and the lower extremities became œdematous. In this state he came into the hospital, on the 21st of April. The breathing was now very much embarrassed—cough not frequent—expectoration easy—the respiratory murmur free from the slightest degree of rattle—percussion of the chest elicited a clear sound, excepting in the region of the heart, which shewed a marked protuberance, 6 inches in extent. The pulse was 120, but regular—the lower extremities œdematous. No thirst. The diarrhœa had ceased—the intellectual functions were quite free. He was bled to 16 ounces, and ptisanes, with nitre and digitalis, exhibited. The night was tranquil, and the next day (17th of the disease) the pulse fell to 70 in the minute, and regular. The œdema of the lower limbs had disappeared—and there was considerable diminution of the præcordial saliency. There was a partial return of sonoriety in the region of the heart. By the 25th day the protuberance had entirely disappeared—that part was nearly as sonorous as any other—the cough and dyspnœa had ceased. The patient soon afterwards left the hospital well. That this was an inflammation of the pericardium, and not of the heart itself, M. Louis thinks is proved by the absence of palpitation—and by the dull sound, owing to effusion into the cavity of the pericardium. This was the second time that our author had observed a protuberance in the region of the heart during pericarditis. In the other case, the fatal termination proved the nature of saliency—in the present, he has no doubt that the same effusion obtained. The œdema of the lower extremities, he remarks, is a phenomenon well worthy of attention. It is almost peculiar, in acute diseases, to affections of the circulating system.

*Case* 2. A young man, 21 years of age, well formed, and of tolerably good constitution, had been ill eight days before admittance into the hospital, on the 4th of June. Two weeks previously he had received a blow on his back, between the right scapula and the spine. A shiver was the first symptom he perceived, which recurred in the evening of the same day. Palpitation, with some dyspnœa, ensued, the latter being, however, habitual. *5th June*, ninth of the disease. Slight oppression—could lie without any additional elevation of the head—pulse calm, regular—heat natural—some palpitation—chest perfectly sonorous, except in the præcordial region, where it was quite dull. It was the same with the respiratory murmur, which was entirely absent in the part that sounded dull. Diluents, with nitre and digitalis. The rigors recurred on the 5th, 7th, 8th, and 9th. Venesection was performed on the 8th, the blood being slightly buffed. Between the 7th and the 25th of June, when the patient quitted the hospital, the following phenomena were noted. The præcordial region gradually regained its sonoriété—so that, by the 12th of June, it was natural. The palpitation did not quite subside till the 22d of the month, which was partly attributable to some errors of diet. The pulse was always regular, and not accelerated. He had no prostration of strength—no œdema of the limbs—no protuberance of the præcordial region. He left the hospital on the 27th day of the malady, quite well.

M. Louis thinks that, although there was no excitement of any consequence in this case, nor saliency of the cardiac region, nor œdema of the limbs, as in the first case; yet that the dull sound and want of respiration in the region of the heart, at the beginning, with the gradual return of sound and respiration in the part—coupled with the palpitation, proved that pericarditis had existed, as there was no evidence of any affection of the lungs. We conceive that, if pericarditis did obtain in this instance, it must have been very slight indeed.

Another case, somewhat similar to the last, is related by M. Louis, but we do not deem it necessary to quote it. The absence of sound and respiration, with some irregularity of the pulse and febrile movement, all gradually disappearing, authorized him, he conceived, to pronounce the disease pericarditis. He conceives that this inflammation is much more frequent, and much less dangerous, than is generally imagined—that like pleuritis, it is always accompanied by more or less effusion, producing the phenomena above-mentioned—and that, when we find adhesion of the pericardium to the heart, we see an instance of pericarditis cured, perhaps without any remedial measures having been used. These observations we commit to the consideration of our readers. But to return to Dr. Elliotson.

He informs us that the treatment of the disease in question does not come within the range of object in these lectures; at the same time, he lets fall some valuable hints relative to the METHODUS MEDENDI. These we shall quote, as they may not prove unacceptable to our readers.

"I have observed free local bleeding more serviceable than general; and that mercury is of equal efficacy in acute pericarditis as in other acute inflammations, over which, wherever they may be situated, a very extensive experience of many years has fully satisfied me, conformably with the observations of so many able physicians, that it possesses far, very far, more power than any other medicine. Bleeding and other ordinary measures cure cases of severe inflammation every day, and, in cases of little danger, may be relied upon. But they frequently fail in cases of intensity; and I know that if, in addition to suitable bleeding, mercurial ptyalism is quickly induced, active inflammation will very rarely destroy; and that, not only is fatality almost always prevented, but far less bleeding is required. This has been my practice from the commencement of my professional life, and I have never met with a necessity for those frightful bleedings of quart after quart, recorded from time to time in our publications, where I also employed mercury with freedom. I have given the antimonium tartarizatum in quantities of a scruple and half a drachm every twenty-four hours, hydrocyanic acid, and other medicines recommended by the Italians, but found them all greatly inferior to mercury. Among the best unquestionably is colchicum, and its power over active gout and rheumatism of the extremities is universally acknowledged to be very great. After the violence of acute pericarditis is subdued, it appears of use in restraining the morbid irritability which sometimes still continues in the heart; and several chronic cases, of which I had despaired, have gradually recovered under perseverance in its use for many months." 11.

The following passages will bear on the subject of pericarditis as viewed by M. Louis.

"The quantity of fluid at a certain period of acute pericarditis, and in chronic pericarditis, is occasionally, but not often, so considerable that hydrops pericardii exists. The quantity should certainly be half a pint for us to expect inconvenience. Unless it is considerable, it is indicated with no more certainty by percussion and auscultation than by the ordinary symptoms. But if the quantity is large, there is a dull sound to a great extent on striking the cardiac region; the heart's action may be perceived very faintly, and perhaps in a diffused manner, so that the epigastrium pulsates or vibrates and may appear fuller than in health; patients have experienced a sense of weight in the cardiac region, and even fluctuation has been detected. In cases of copious effusion of blood, pus, or serum, into the pericardium, these symptoms have suddenly appeared, and good examples of the occurrence of some of them may be found in Andral.

A fluid occasionally collects in the pericardium, as in the pleura and peritoneum, arachnoid and tunica vaginalis, by a slow process, not amounting to inflammation. The membrane is not red, but perhaps opake, and even thickened and of a satin whiteness. This condition is, I believe, where no redness of inflammation is visible, the common cause of ascites, chronic hydrocephalus, hydrocele, and idiopathic hydrothorax; and though, like the state which gives birth, as I shall presently mention, to one kind of adhesions in serous membranes, it may be the result of a change allied to inflammation, it hardly merits the title of inflammation, from the absence of inflammatory symptoms, the absence of redness in the membrane, the pellucidity of the fluid, and the inutility of anti-inflammatory measures.

The adhesions within the pericardium left after pericarditis are almost the only instances that occur in this membrane. I have never seen adhesions except with redness of the membrane, or the presence of turbid fluid, or after the existence of decided symptoms of pericarditis. In the pleura they are continually found, without the least previous symptoms of inflammation, and without redness or turbid effusion; and though, like chronic dropsy of the membranes, they may result from what cannot be proved to be an inflammatory state, they certainly, like it, are no proofs of any thing deserving the decided name of inflammation.

In the case neither of the pleura nor pericardium, do they in general produce the slightest inconvenience. I have seen the whole pericardium so coherent that its cavity was entirely abolished, and yet the symptoms which had been present were exactly commensurate with the organic disease of the heart which existed at the same time, and had certainly no relation to the adhesion. I cannot say I ever observed a symptom produced, except in one case, and there a single thick adhesion extended along the front of the heart. In the supine posture this must have been dragged down by the subjacent heart, and must have tended to drag the pericardium of the front of the chest with it, and to suspend the heart, so that the parietal and cardiac pericardium at their points of union with it must have put upon the stretch. The patient accordingly had been unable to lie on her back, on account of a smarting pain produced in this posture at the front of the cardiac region. Bertin,* in his excellent work upon diseases of the heart, states that adhesions often produce no symptoms, but gives one case in illustration that inconvenience sometimes is felt: yet in this case, adduced singularly enough, the substance of the heart, in addition to the adhesions, was found very soft—a change quite sufficient to explain every symptom that occurred." 11.

To the second lecture, embracing a very distinct and very important subject—"affections of the lining membrane of the heart," we must dedicate another article. Such practitioners as Cooper and Elliotson do not come every day under review, and as their works are in forms that render them but little accessible to the great mass of the profession, we must endeavour to supply the loss thus sustained, by more copious and careful analyses.

---

* Traité des Maladies du Cœur et des Gros Vaisseaux, par R. J. Bertin. Paris, 1824, p. 254.

